# Inline perfusion mapping provides insights into the disease mechanism in hypertrophic cardiomyopathy

**DOI:** 10.1136/heartjnl-2019-315848

**Published:** 2019-12-10

**Authors:** Claudia Camaioni, Kristopher D Knott, Joao B Augusto, Andreas Seraphim, Stefania Rosmini, Fabrizio Ricci, Redha Boubertakh, Hui Xue, Rebecca Hughes, Gaby Captur, Luis Rocha Lopes, Louise Anne Elizabeth Brown, Charlotte Manisty, Steffen Erhard Petersen, Sven Plein, Peter Kellman, Saidi A Mohiddin, James C Moon

**Affiliations:** 1 Advanced Cardiac Imaging, Barts Health NHS Trust, London, UK; 2 Institute of Cardiovascular Science, University College London, London, UK; 3 University of Chieti-Pescara, Chieti, Italy; 4 The William Harvey Research Institute, Queen Mary University of London, London, UK; 5 National Institutes of Health, Bethesda, Maryland, USA; 6 Department of Biomedical Imaging Science, University of Leeds, Leeds, UK

**Keywords:** Hypertrophic cardiomyopathy, Advanced cardiac imaging, Cardiac magnetic resonance (CMR) imaging

## Abstract

**Objective:**

In patients with hypertrophic cardiomyopathy (HCM), the role of small vessel disease and myocardial perfusion remains incompletely understood and data on absolute myocardial blood flow (MBF, mL/g/min) are scarce. We measured MBF using cardiovascular magnetic resonance fully quantitative perfusion mapping to determine the relationship between perfusion, hypertrophy and late gadolinium enhancement (LGE) in HCM.

**Methods:**

101 patients with HCM with unobstructed epicardial coronary arteries and 30 controls (with matched cardiovascular risk factors) underwent pixel-wise perfusion mapping during adenosine stress and rest. Stress, rest MBF and the myocardial perfusion reserve (MPR, ratio of stress to rest) were calculated globally and segmentally and then associated with segmental wall thickness and LGE.

**Results:**

In HCM, 79% had a perfusion defect on clinical read. Stress MBF and MPR were reduced compared with controls (mean±SD 1.63±0.60 vs 2.30±0.64 mL/g/min, p<0.0001 and 2.21±0.87 vs 2.90±0.90, p=0.0003, respectively). Globally, stress MBF fell with increasing indexed left ventricle mass (R^2^ for the model 0.186, p=0.036) and segmentally with increasing wall thickness and LGE (both p<0.0001). In 21% of patients with HCM, MBF was lower during stress than rest (MPR <1) in at least one myocardial segment, a phenomenon which was predominantly subendocardial. Apparently normal HCM segments (normal wall thickness, no LGE) had reduced stress MBF and MPR compared with controls (mean±SD 1.88±0.81 mL/g/min vs 2.32±0.78 mL/g/min, p<0.0001).

**Conclusions:**

Microvascular dysfunction is common in HCM and associated with hypertrophy and LGE. Perfusion can fall during vasodilator stress and is abnormal even in apparently normal myocardium suggesting it may be an early disease marker.

## Introduction

Hypertrophic cardiomyopathy (HCM) affects 1 in 500 people and is characterised clinically by unexplained hypertrophy and genetically by mutations in genes encoding (primarily) sarcomeric proteins.^1 2^ The histological features are myocyte disarray, left (±right) ventricular hypertrophy (LVH), small vessel disease and fibrosis, but how these features develop and relate to adverse outcomes is poorly understood.

Advanced cardiac imaging with echocardiography, myocardial perfusion scintigraphy (MPS), cardiovascular magnetic resonance (CMR) and positron emission tomography (PET) can measure hypertrophy, microvascular dysfunction and fibrosis. However, quantification of these processes is not well integrated into clinical care and this may partially explain our lack of progress on developing disease-modifying therapies.

Ischaemia in HCM is likely a key disease pathway. Chest pain is frequent, ischaemic ECG changes are common^3^ and sudden cardiac death (SCD) is relatively more common during exercise.^4^ Several mechanisms may contribute to ischaemia in HCM including small vessel abnormalities, demand-supply mismatch due to hypertrophy, reduced perfusion pressure related to shortened diastolic time, high diastolic pressure, left ventricular outflow tract (LVOT) obstruction and possibly myocardial bridging.^5–8^

Microvascular dysfunction in HCM was first studied by nuclear medicine techniques, demonstrating perfusion impairment even in apparently minimally affected segments,^9^ with ischaemia correlating with hypertrophy and poor outcomes.^10 11^ CMR has demonstrated evidence of coronary microvascular flow impairment,^12^ finding inverse correlations between perfusion and both hypertrophy and fibrosis.^13 14^ However, the quantification techniques used in these studies have been highly labour intensive in terms of both image acquisition and analysis. Consequently, quantitative perfusion has been less actively explored than scar as a candidate risk factor for SCD and has been outside of the realm of clinical care.

**Figure 1:**
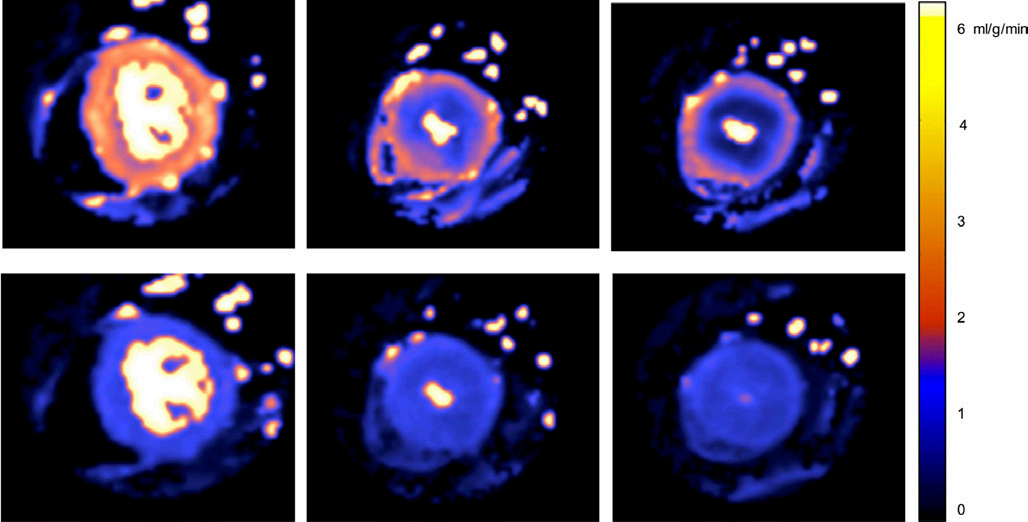
Perfusion maps. Base, mid and apical left ventricular slices (left to right) at peak stress (top) and rest (bottom) in a patient with apical hypertrophic cardiomyopathy. During stress, a circumferential mid to apical perfusion defect is observed, more severe at the apex, particularly in the endocardial layer where the stress myocardial blood flow (MBF) is lower than the rest. *Stress MBF values*: basal 1.51 mL/g/min, mid-ventricular 0.82 mL/g/min and apical 0.53 mL/g/min. *Rest MBF values*: basal 0.93 mL/g/min, mid-ventricular 0.79 mL/g/min and apical 0.77 mL/g/min.

**Figure 2:**
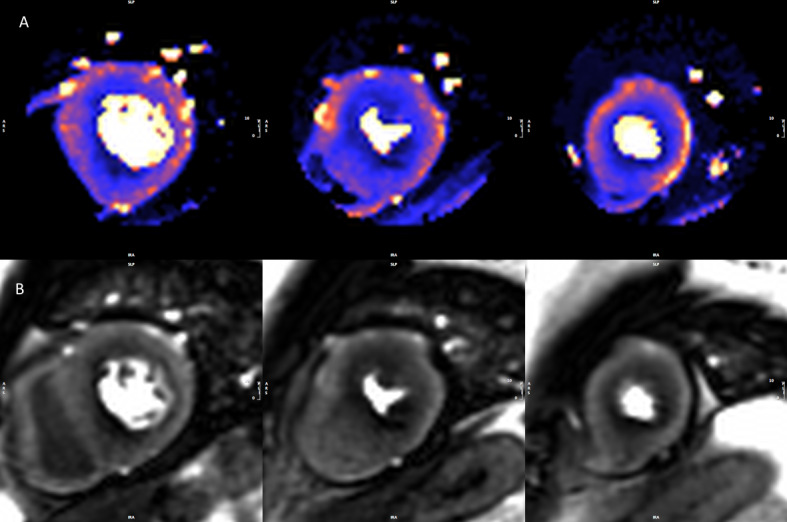
Stress perfusion maps and raw perfusion images. Example of a set of perfusion maps (A) and raw perfusion images (B) from a patient with hypertrophic cardiomyopathy. The perfusion defect is appreciated on visual read but the full extent of hypoperfusion is more readily seen on the perfusion maps. Global stress myocardial blood flow (MBF) is 1.38 mL/g/min.

Recent advances in CMR perfusion mapping now permit high-resolution, pixel-wise myocardial blood flow (MBF) quantification automatically and inline at the scanner (figures 1 and 2) 15 using the Gadgetron software framework.^16^ In suspected ischaemic heart disease the technique has been validated using PET, angiography and invasive fractional flow reserve (FFR).^17–19^ Perfusion mapping has also provided insights into the disease process in Fabry disease.^20^ CMR has further advantages as it does not use ionising radiation, has higher spatial resolution than other imaging modalities and is becoming more widely available.

We sought to retrospectively quantify myocardial perfusion in clinically referred patients with HCM to better understand the relationship between perfusion, hypertrophy and LGE in the disease process. We hypothesised that perfusion in HCM would be related to markers of disease severity such as hypertrophy and LGE and that impaired perfusion may precede hypertrophy in some myocardial segments suggesting it is an early marker of disease.

## Methods

### Study design and population

Patients with HCM referred for stress CMR as part of standard clinical care at Barts Heart Centre (London, UK) were enrolled between June 2016 and June 2019. The diagnosis of HCM had been made previously based on conventional diagnostic criteria according to the European Society of Cardiology guidelines.^2^ Patients were excluded if the they had epicardial coronary artery disease, defined as >50% diameter stenosis in a major coronary artery, by either invasive coronary angiography (56%) or CT (44%) within 3 months of CMR and compared with age, gender, body surface area (BSA) and cardiovascular risk factor matched controls.

Controls included a matched chest pain cohort referred for perfusion CMR. These were patients referred with cardiovascular risk factors (excluding history of myocardial infarction) and atypical chest but otherwise normal CMR (normal structure, function, no perfusion defect and no LGE). This cohort was used to control for other risk factors of microvascular dysfunction which might impair perfusion in patients with HCM independent of the HCM disease process. Study exclusion criteria were contraindications to CMR, adenosine or gadolinium. This study was performed in accordance with the principles of the Declaration of Helsinki and all participants gave written informed consent (217 671 and/or 14/EE/0007).

### Image acquisition

CMR was performed using either a Magnetom Aera 1.5T or Prisma 3.0T system (Siemens Healthineers, Erlangen, Germany). A standard CMR protocol was used including cine images, stress and rest perfusion and late gadolinium enhancement.^21^ All subjects abstained from caffeine for at least 12 hours. Adenosine was infused for 4 min at 140 µg/kg/min (increased to 175 µg/kg/min if there was no heart rate response and symptoms). At peak vasodilator stress a gadolinium-based contrast agent (Dotarem, Guerbet, Paris, France) was injected at a dose of 0.05 mmol/kg at a rate of 4 mL/s. Three short axis slices (base, mid and apex) were acquired during the first pass of contrast (60 measurements). The acquisition was repeated at rest, with the short axis cine stack acquired between stress and rest.

Perfusion mapping was performed automatically and inline as previously described.^15^ In brief, this was a single-bolus, dual-sequence technique with a balanced steady-state free precession (bSSFP) pulse sequence readout. LGE images were acquired in long axis and short axis using a free-breathing bright blood single-shot bSSFP sequence with phase-sensitive inversion recovery reconstruction and motion correction. Sequence details are provided in the supplementary appendix.

### Image analysis

**Figure 3:**
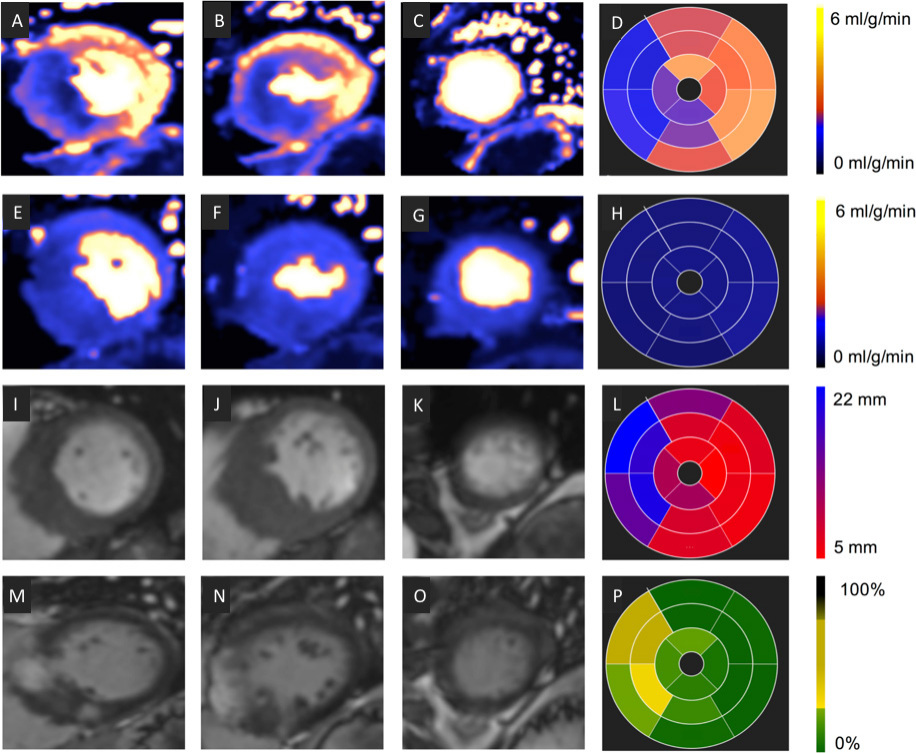
Image analysis. Correlation among stress myocardial blood flow (MBF; A–D), rest MBF (E–H), wall thickness (WT) (I–L) and late gadolinium enhancement (LGE) (M–P). Each row shows a short-axis view (from left, base-mid-apex) and the corresponding 16 segment bullseye. Values are expressed using a specific colour look-up table for MBF (D, H), WT (L) and LGE where value is percentage of enhanced pixels per segment (P).

**Figure 4:**
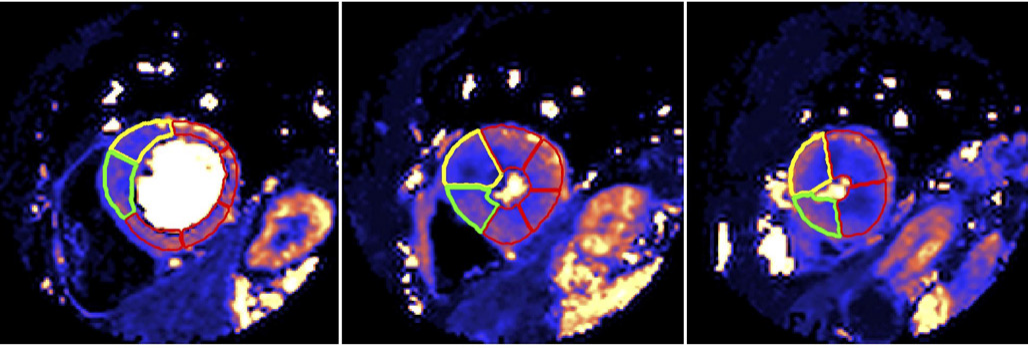
Perfusion map automatic segmentation using machine learning. Basal, mid and apical (left to right) short-axis left ventricular (LV) slices demonstrating automatic segmentation using machine learning. The yellow segments are the starting point for each slice (ie, segments 1, 7, 13) and the green segments are the second segment in each slice, allowing easy quality control. The remaining segments are contoured red.

Offline analysis was performed using commercial software (cvi42, Circle Cardiovascular Imaging, Canada). The raw perfusion images were scored for the presence or absence of visual perfusion defects. Cine images, LGE and perfusion maps were analysed globally and segmentally according to the American Heart Association (AHA) 17 segment model (minus the apical cap, figure 3).^21^ The maximum end-diastolic wall thickness (excluding papillary muscles) was determined per segment and the maximum wall thickness globally listed. LGE was quantified using the 5 SD technique, where each slice is manually contoured and a region of interest is drawn in the ‘remote’ myocardium. The amount of LGE was calculated per segment and globally. Subsequently, each segment was scored as having visually confluent, diffuse or no LGE. Global (average across all pixels throughout the myocardium) and segmental MBFs (average of all pixels within each segment) were calculated inline from the perfusion maps (where each pixel encodes MBFs in mL/g/min). For each slice, the endocardial and epicardial borders were contoured automatically using a machine learning approach (figure 4). The myocardial perfusion reserve (MPR) was calculated as the ratio of stress to rest MBF. Each segment was also divided into endocardial (inner 50%) and epicardial (outer 50%) regions. Each component of the analysis was performed by two independent observers blinded to other CMR parameters.

### Statistical analysis

Statistical analysis was performed using SPSS (V.25, IBM). Categorical data were presented as frequencies and percentage, continuous as mean±SD or median and IQR as per normality. The Student’s t-test was used for parametric data, Mann-Whitney U test for non-parametric and χ^2^ for categorical variables. Regression analysis was used to determine the factors associated with perfusion in the HCM subjects. The analysis was performed on both a global (subject level) and segmental level (where the effect of wall thickness and LGE on perfusion was assessed on a segment basis). A mixed effects linear regression model was used at the segmental level to control for within-subject dependency (subjects included as the random effect).

## Results

### Population

In total, 101 patients with HCM (male 82%, mean age 49.7±12.1) and 30 patient controls (male 77%, mean age 51.5±14.1, p=0.60 and p=0.48, respectively). The HCM clinical phenotype was asymmetric septal hypertrophy in 80 (79.2%), concentric in 13 (12.9%) and apical or apical predominant in 8 (7.9%). No patient had undergone septal reduction therapy (myectomy or alcohol ablation) and no patients had a cardiac implantable electronic device. Seventy-one patients (70.3%) were scanned at 1.5T and 30 (29.7%) were scanned at 3.0T.

Compared with the control cohort, the patients with HCM had higher indexed left ventricle (LV) mass, maximum wall thickness and ejection fraction (EF) (table 1). There was no difference in end-diastolic volume (EDV). Forty-nine patients with HCM had LGE (48.5%) and there were perfusion defects in 79 (78.2%) on clinical read. In the HCM group, LVOT obstruction was present under scanning conditions in 17 (16.8%).

**Table 1:** Characteristics of patients with hypertrophic cardiomyopathy (HCM) and controls

	HCM n=101	Controls n=30	P value
Age (years)	49.7±12.1	51.5±14.1	0.48
Male, n (%)	82 (82)	23 (77)	0.60
BSA (m^2^)	2.03±0.26	1.97±0.21	0.32
Diabetes, n (%)	17 (17)	7 (23)	0.43
Hypertension, n (%)	44 (44)	12 (40)	0.83
Dyslipidaemia, n (%)	21 (21)	7 (23)	0.80
LVEDVi (mL/m^2^)	72.9±14.1	77.1±19.8	0.19
LVEF (%)	74.1±7.7	64.8±9.3	**<0.001**
LV mass indexed (g/m^2^)	87.0±28.3	55.0±13.0	**<0.001**
LGE, n (%)	49 (49)	0 (0)	**<0.001**
Stress MBF (mL/g/min)	1.62±0.60	2.31±0.64	**<0.001**
Rest MBF (mL/g/min)	0.79±0.24	0.82±0.25	0.47

Data are presented as mean±SD unless stated. P values in bold are statistically significant.

BSA, body surface area; LGE, late gadolinium enhancement; LVEDVi, left ventricle end-diastolic volume indexed for BSA; LVEF, left ventricular ejection fraction; MBF, myocardial blood flow.

### Quantitative perfusion analysis

**Figure 5:**
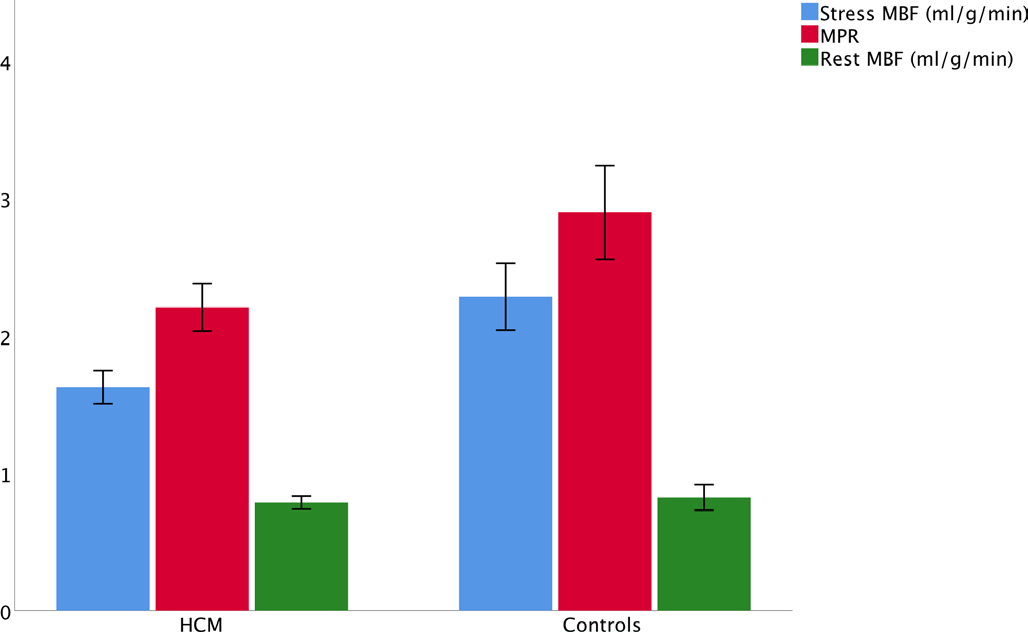
Global perfusion analysis. Differences in stress mean myocardial blood flow (MBF, blue), myocardial perfusion reserve (MPR, red) and rest MBF (green) between patients with hypertrophic cardiomyopathy (HCM) and controls. The bars display the 95% CIs. Stress MBF and MPR were lower in HCM than controls (1.63±0.60 vs 2.30±0.64 mL/g/min and 2.21±0.87 vs 2.90±0.90, respectively, both p<0.0001). There was no difference in rest MBF (0.79±0.24 and 0.82±0.24, respectively, p=0.47).

Global stress MBF and global MPR were lower in HCM than controls (MBF 1.63±0.60 mL/g/min vs 2.30±0.64 mL/g/min, p<0.001; MPR 2.21±0.87 vs 2.90±0.90, p<0.001) with no difference at rest (0.79±0.24 and 0.82±0.24, p=0.47) (figure 5). Although 78% of patients with HCM had a perfusion defect on visual analysis, there was no difference between the global stress MBF of those with a perfusion defect and those with no perfusion defect (1.61±0.59 mL/g/min vs 1.70±0.66 mL/g/min, p=0.522). There was no difference in global perfusion in patients scanned at 1.5T vs 3T (1.61 mL/g/min vs 1.68 mL/g/min, respectively, p=0.552).

In HCM, stress MBF was lower in the subendocardium versus subepicardium (1.38±0.57 mL/g/min vs 2.32±0.97 mL/g/min, p<0.001), but rest not significantly different (figures 1 and 2). This stress developed transmural gradient also occurred in controls (2.20±0.61 mL/g/min vs 3.24±0.94 mL/g/min, p<0.001).

A multivariate linear regression analysis was performed to see the factors contributing to perfusion in patients with HCM on a global (whole heart) basis. Included in the regression were demographic factors (age, sex), comorbidities (hypertension, diabetes mellitus and dyslipidaemia) and CMR parameters (total LGE, EDV and mass indexed for BSA and EF). Of these, only increasing indexed LV mass was the only factor associated with reduced perfusion (R^2^ for the model 0.186, p=0.036, table 2) suggesting that reduced perfusion is mainly independent of currently measured parameters.

**Table 2:** Multiple linear regression model for the dependent variable global stress myocardial blood flow (MBF). Global stress MBF was independently influenced by indexed left ventricle (LV) mass

	Beta	SE	95% CI lower bound	95% CI upper bound	P value
Constant	3.317	0.939	1.439	5.184	**0.001**
Age	−0.009	0.006	−0.210	0.003	0.135
Sex	−0.101	0.176	−0.451	0.229	0.569
Diabetes	0.138	0.168	−0.196	0.472	0.413
Hypertension	−0.136	0.130	−0.396	0.123	0.299
Dyslipidaemia	−0.264	0.161	−0.584	0.057	0.106
LVEDVi	0.003	0.006	−0.009	0.014	0.653
LVEF	−0.012	0.009	−0.029	0.006	0.193
LV mass-i	−0.006	0.003	−0.013	0.000	**0.044**
LGE	0.005	0.004	−0.002	0.012	0.170

R^2^=0.186 for the model, p=0.036. P values in bold are statistically significant.

LGE, late gadolinium enhancement; LVEDVi, left ventricle end-diastolic volume indexed for body surface area (BSA); LVEF, left ventricular ejection fraction; LV mass-i, left ventricle mass indexed for BSA.

Analysis was also performed for each myocardial segment. On a per-segment basis, the parameters influencing stress MBF were determined using a multivariable linear regression model taking account within subject dependency. Both the percentage of myocardial late enhancement and wall thickness were associated with impaired MBF per segment (p<0.001 for both, table 3). A further segmental analysis was performed comparing stress MBF in apparently ‘normal’ segments in the HCM cohort to the control group. When only LGE-free segments with a wall thickness <11 mm were compared with controls, the stress MBF remained significantly lower (1.88±0.81 mL/g/min vs 2.32±0.78 mL/g/min, p<0.001).

**Table 3:** Mixed effects linear regression model, controlling for within-subject dependency, for the dependent variable segmental stress myocardial blood flow (MBF)

	Beta	SE	95% CI lower bound	95% CI upper bound	P value
Intercept	2.269	0.070	2.134	2.409	**<0.001**
Wall thickness	−0.050	0.004	−0.060	−0.043	**<0.001**
LGE	−0.006	0.001	−0.008	−0.004	**<0.001**

Wall thickness and percentage late gadolinium enhancement (LGE) per segment were treated as continuous variables and were independently associated with stress MBF. P values in bold are statistically significant.

In HCM, stress flow paradoxically lower than rest (MPR <1) was observed in at least one AHA segment in 21 patients (21%) (example in figure 1). The finding was more common with increasing wall thickness and LGE (only 1.8% of segments with MPR <1 had normal wall thickness and no LGE). An MPR <1 was mainly a subendocardial phenomenon (subendocardial, transmural, subepicardial: 16.3%, 5.6% and 4.7% of segments) meaning a subendocardial MPR <1 occurred in at least one segment in 43 (42.6%) patients.

Segments with visually confluent LGE had a mean stress MBF 1.31 mL/g/min, segments with diffuse LGE 1.38 mL/g/min, segments with LVH but no LGE 1.48 mL/g/min and segments with no LGE or LVH 1.88 mL/g/min. There was no significant difference in the stress MBF of confluent versus diffuse LGE (p=0.352). Confluent LGE had lower stress MBF than LVH segments without LGE (p=0.015) but this did not reach significance for diffuse LGE (p=0.054).

## Discussion

In this largest quantitative CMR perfusion study published in HCM to date, we have shown that microvascular dysfunction is common and somewhat underappreciated in HCM with 78% of patients having perfusion defects on clinical read. Also, global MBF was low throughout the population. The perfusion abnormalities are not explained by epicardial coronary disease or conventional cardiovascular risk factors, and only partly explained by LGE and hypertrophy, occurring even in the absence of both. Stress MBF is however lowest in the most hypertrophied and fibrotic segments and perfusion can actually fall during stress (giving an MPR <1). This was a relatively common finding, with over one-fifth of patients having at least one myocardial segment with a lower stress blood flow.

Our results support the previous literature of perfusion in HCM using different modalities over decades.^9 11–14 22–24^ Initial (non-quantitative) assessment was performed using MPS. O’Gara *et al* in 1987 found visual stress-induced perfusion defects in 41/72 (57%) patients with HCM.^25^ The perfusion defects seen were independent of patients’ symptoms. Furthermore, visual perfusion defects have been shown to be associated with an abnormal blood pressure response to exercise^26^ and to improve with medical therapy with verapamil.^27^ In our study, the per cent of patients with perfusion defect was higher than this at 78%. This may reflect improvements in sensitivity in detection of perfusion defects using latest CMR technology or the fact that this was a clinically referred patient population.

Quantitative perfusion using PET has also investigated MBF in HCM. Camici *et al* investigated 23 patients with HCM and found that they had impaired perfusion reserve compared with a comparator cohort, even in the non-hypertrophied LV-free wall.^9^ There is also prognostic information encoded in MPR as determined by PET and those with impaired perfusion have worse outcomes.^11^

The evidence base for quantitative perfusion CMR is more limited but increasing in recent years. Petersen *et al* investigated 35 patients with HCM finding impairment in stress MBF correlating with fibrosis and wall thickness predominately affecting the subendocardium. These findings are consistent with what we have found. However, we additionally found that perfusion abnormalities could even be present in segments that were non-hypertrophied and contained no LGE.^12 13^ To our knowledge, this is the largest CMR quantitative perfusion study, with prior cohorts including 30–40 patients and similar to the largest quantitative PET study of 100 patients.^28^

Ismail *et al* investigated 35 patients with HCM using a pixel-wise quantification approach with similar findings.^13^ As well as being a larger cohort, perfusion techniques have developed, here using the single-bolus, dual-sequence approach to overcome arterial input function clipping, automated motion correction requiring no uncomfortable breath holds, inline map reconstruction and automated flow quantification (within 30 s) with no user input for global, regional or subsegmental flow data extraction.^15^ Other small HCM cohorts have used either semiquantitative or the dual-sequence approach with broadly consistent results.^14 24^ We found an inverse correlation between MPR or stress MBF and both wall thickness and LGE. However, these associations were relatively modest, and even myocardial segments without LVH or LGE had impaired perfusion, suggesting that microvascular dysfunction is substantially independent of macroscopic scar and hypertrophy, and may be an important marker in early HCM. There have been pathological studies looking at ischaemia in HCM. Basso *et al* looked at 19 patients with HCM with SCD.^29^ They found abnormal intramural small vessels in the myocardium and evidence of all stages of ischaemia from acute to chronic appearances even in the absence of epicardial coronary artery disease, and postulated that HCM replacement fibrosis is triggered by myocardial ischaemia with combination of disarray and ischaemia being the arrhythmic substrate. In another autopsy study of 72 patients Varnava *et al* found a poor correlation between small vessel disease and LVH and there was evidence of small vessel disease even in children.^5^

A striking finding was that stress flow in HCM could be paradoxically lower than rest in HCM. A perfusion reserve <1 has previously been observed and thought to be related exclusively to scar^14^ or modelling artefact. Here, it mostly occurred in the endocardial layer (16.3% of all myocardial segments) and was associated with increased LGE and wall thickness. However, LGE and hypertrophy cannot fully explain the phenomenon and a small per cent (1.8% transmural or 6.9% subendocardial segments) of this MPR <1 segment was free from LGE and LVH. There are multiple possible explanations for perfusion falling during vasodilator stress. Macroscopic steal appears unlikely as the areas of hypoperfusion are large but microscopic steal remains possible. Another explanation would be an altered myocardial vasomotor response to adenosine in HCM. The vascular response to adenosine is organ specific (eg, splenic vasoconstriction, myocardial vasodilatation) and modification of the myocardium in disease could play a role. Another possibility would include a mechanical explanation with prolonged regional systole and altered myocardial mechanics secondary to a vasodilatation-induced tachycardia inducing perfusion defects.^7^

In this study we have demonstrated a transmural perfusion gradient in subjects at stress, where the subendocardial perfusion is reduced relative to the subepicardium. This is consistent with previous microsphere studies using animal models^30 31^ and CMR studies in health and disease.^32 33^ In contrast, PET studies have suggested that at stress the subendocardium is more highly perfused than the subepicardium.^34 35^ Possible explanations include differences in the spatial resolution of the techniques or other method-specific factors which influence the perfusion data for PET and CMR.

Our study is limited by the fact that our patient population was clinically referred for scans which may put them into a higher risk category. Additionally, all were recruited from a tertiary centre that includes specialist cardiomyopathy services. Similar to other studies, there was no tissue correlation and our cohort was not designed for prognostic endpoints. Vasodilator stress demonstrates areas of hypoperfusion and is one step remote from ischaemia but the link between these concepts has been made previously.

In conclusion, using fully quantitative CMR perfusion mapping incorporated into a clinical workflow, we have demonstrated the role of microvascular dysfunction in HCM and that while this is statistically strongly associated with regional hypertrophy and fibrosis, these processes explain only a small amount of myocardial stress blood flow heterogeneity. Flow was noted to actually fall during vasodilator stress and can be abnormal even in remote (no LVH, no LGE) myocardium suggesting microvascular dysfunction may occur early in phenotype development. CMR perfusion mapping is a useful new tool to investigate the pathophysiology of cardiomyopathy, making evaluating ischaemia a testable SCD risk factor and a potential therapeutic target.

**Key messages**

**What is already known on this subject?**

- Hypertrophic cardiomyopathy (HCM) is a common genetic cardiomyopathy in which there is myocyte disarray, left ventricular hypertrophy, small vessel disease and hypertrophy.

**What might this study add?**

- This study has shown that microvascular dysfunction is common in HCM and worsens with increasing disease severity (hypertrophy and fibrosis). Perfusion can actually fall with vasodilator stress and can be abnormal even in apparently normal myocardium suggesting it may occur early in the disease process.

**How might this impact on clinical practice?**

- Quantitative perfusion cardiovascular magnetic resonance is becoming routine in clinical practice and may be useful in detecting early disease in HCM, could act as a testable risk factor for sudden cardiac death and be a potential therapeutic target.

10.1136/heartjnl-2019-315848.supp1 Supplementary data
