# Supplementary material for: Inline perfusion mapping provides insights into the disease mechanism in hypertrophic cardiomyopathy
Source: Heart. 2019 Dec 10;106(11):824–9. doi: 10.1136/heartjnl-2019-315848 (PMC7282549; doi:10.1136/heartjnl-2019-315848)
Supplement: Supplementary data [file heartjnl-2019-315848supp001.pdf]

Appendix

CMR sequence parameters:

Supplementary table 1. AIF sequence parameters

Sequence	FLASH
TE	0.76 & 1.76ms
TR	2.45ms
FA	5 degrees
Matrix	64x48
FOV (typical)	360x270x10mm^3
PE order	Linear
Parallel imaging	TPAT3
TI	23.8ms
SR preparation	6-pulse
Imaging duration	42ms
Total duration	68.2ms

Abbreviations: FLASH – fast low angle shot, TE – echo time, TR - repetition time, FA – flip angle, FOV – field of view, PE – phase encoding, TI – inversion time, SR- saturation recovery

Supplementary table 2. Myocardial perfusion sequence parameters.

Sequence	SSFP
TE	1.04ms
TR	2.5ms
FA	50 degrees
Matrix	192x111
FOV (typical)	360x270x8mm^3
PE order	Linear
Partial fourier	3/4
Parallel imaging	TPAT3
TS	105ms
SR preparation	5-pulse
Imaging duration	70ms
Total duration	142ms/slice

Abbreviations: SSFP – steady state free precession, TE – echo time, TR - repetition time, FA – flip angle, FOV – field of view, PE – phase encoding, TS – saturation delay, TD – trigger delay, SR- saturation recovery.
